# Chart validation of an algorithm for identifying hereditary progressive muscular dystrophy in healthcare claims

**DOI:** 10.1186/s12874-019-0816-7

**Published:** 2019-08-09

**Authors:** Xiaoxue Chen, Abiy Agiro, Ann S. Martin, Ann M. Lucas, Kevin Haynes

**Affiliations:** 1 HealthCore, Inc, 123 Justison St, Suite 200, Wilmington, DE 19801 USA; 2 Parent Project Muscular Dystrophy, Hackensack, NJ USA; 3 Sanofi Genzyme, Cambridge, MA USA

**Keywords:** Duchenne, Muscular dystrophy, Validation, Claims analysis

## Abstract

**Background:**

Muscular dystrophies (MDs) are a group of inherited conditions characterized by progressive muscle degeneration and weakness. The rarity and heterogeneity of the population with MD have hindered therapeutic developments as well as epidemiological and health outcomes research. The objective of the study was to develop and validate a case-finding algorithm utilizing administrative claims data to identify and characterize patients with MD.

**Methods:**

This retrospective cohort study used medical chart validation to evaluate an ICD-9/10 coding algorithm in a large commercial claims database. Patients were identified who had ≥2 office visits with a diagnosis of hereditary progressive MDs from January 1, 2013 through December 31, 2016, were male, and younger than 18 years at the time of first MD diagnosis. Cases who met the algorithm were then validated against medical charts. Diagnoses of MD and specific type (Duchenne, Becker, or other MD) were confirmed by medical chart review by trained reviewers. Positive predictive value (PPV) and 95% confidence intervals (CI) were calculated using a 2 × 2 contingence table. Patient demographic, clinical, and health utilization characteristics were summarized using basic descriptive statistics.

**Results:**

Charts were obtained and reviewed for 109 patients who met the algorithm. The PPV of the case-identifying algorithm for MD was 95% (95% CI 88–98%). Of the 103 confirmed MD cases, 87 patients (85%, 95% CI 76–91%) had Duchenne or Becker MD; 76 patients (74%, 95% CI 64–82%) had Duchenne MD, and 11 patients (11%, 95% CI 5–18%) had Becker MD. A total of 74 (67.9%) patients had ≥1 pediatric complex chronic condition (other than neurologic/neuromuscular disease); 54 (49.5%) had cardiovascular conditions; 14 (12.8%) had respiratory conditions; 50 (45.9%) had bone-related issues; 11 (10.1%) had impaired growth; and 6 (5.5%) had puberty delay.

**Conclusions:**

The results of this study demonstrate that the case-finding algorithm accurately identified patients with MD, primarily Duchenne MD, within a large administrative database. The algorithm, which was constructed using a few items easily accessible from claims, can be used to facilitate epidemiological and health outcomes research in the Duchenne patient population.

**Electronic supplementary material:**

The online version of this article (10.1186/s12874-019-0816-7) contains supplementary material, which is available to authorized users.

## Background

Muscular dystrophies (MDs) are inherited conditions typically characterized by progressive muscle degeneration and weakness leading to increased disability [1]. There are a number of specific types of MD disorders, including Duchenne and Becker, all of which vary in severity, age of onset, pattern of inheritance, and life expectancy [1].

MDs are rare, with a total combined prevalence of 19.8 to 25.1 per 100,000 person-years [2]. Among them, Duchenne MD is the most common and severe pediatric-onset form. The disease is rapidly progressive, lethal, and has a prevalence of 15.9 to 19.5 per 100,000 person-years [3]. Duchenne MD is caused by mutations in the dystrophin gene that result in absent or insufficient functional dystrophin, leading to progressive muscular damage and degeneration. Symptoms first appear between 1 and 3 years, usually beginning in the calf, pelvis, or thigh muscles [3]. By age 12, most children lose the ability to walk. A different mutation in the same gene results in Becker MD, which has milder and more varied signs and symptoms.

Although there is no cure for any form of MD, medications and therapies help alleviate symptoms and slow disease progression [4]. In recent years, there have been increasing numbers of clinical trials with existing therapies and novel genetic and molecular therapies underway [5, 6]. However, the rarity and heterogeneity of the population with MD have posed challenges to patient recruitments in therapeutic developments [5].

Because of the low numbers of patients who qualify for and engage in trials, studies/initiatives outside clinical trials are much needed to broaden the knowledge of existing glucocorticoid therapies, and validate clinical meaningful and reliable outcome measures to assess emerge therapies, such as patient registries, natural history studies. Claim-based studies are another option. Claims databases from health plans are used to identify patient cohorts for research, offering the advantage of providing large real-world populations at little cost, although diagnostic codes may be inaccurate [7–10]. To overcome the limitation, the Duchenne Registry team at Parent Project Muscular Dystrophy developed the case-finding algorithm used in this study. A similar algorithm was used to identify patients with Duchenne MD in previous studies with large hospital networks. In the case of MD, the *International Classification of Diseases* versions 9 and 10 (ICD-9/10) diagnosis codes have broadly identified the overall population with MD, but did not previously differentiate among MD types. Starting on October 1, 2018 the CMS Addenda included a refined ICD-10 code for Duchenne or Becker MD that distinguishes the dystrophinopathies from the broad categorization. As the new ICD-10 code needs several years to accumulate meaningful length of data, our study explored and validated a case-finding algorithm utilizing large healthcare administrative claims data and characterized the patient cohort under this case-finding algorithm to help assemble a patient cohort and facilitate research from data captured prior to the implementation of the new ICD code.

## Methods

### Study design

This was a retrospective cohort study using medical chart validation to evaluate an ICD-9/10 coding algorithm in a large administrative claims database. The Health Core Integrated Research Database (HIRD®) is a longitudinally integrated database of medical and pharmacy claims and electronic laboratory results from a large commercial health insurer. HIRD contains claims data from 14 commercial health plans geographically dispersed across the United States. An independent institutional review board (IRB) has reviewed the study and granted a waiver of authorization from informed consent to allow access of medical charts.

### Study setting and population

Patients with a diagnosis of hereditary progressive MDs (ICD-9 359.1 or ICD-10 G71.0) between January 1, 2013 through December 31, 2016 (study period) were identified in HIRD. According to the algorithm, patients were required to have at least two office visits with MD diagnosis, to be males and younger than 18 years at the time of the first MD diagnosis. To maximize the amount of information obtained from patient medical charts to ascertain cases, we sampled patients who had a neurologist, primary care physician (PCP) or pediatrician involved in MD care in the chart study. Patients were excluded if they enrolled in Administrative Service Only plans or were not currently active members of plans.

### Case confirmation

The diagnosis of MD and specific type (Duchenne, Becker, or other MD) were confirmed by medical chart review (considered the gold standard) by a third-party medical chart abstraction vendor. Two medical chart reviewers (certified nurses with training) performed chart reviews. As part of the quality control process, the first 10 cases selected by the chart reviewers were also reviewed by two of the authors and a clinical expert. The cases were confirmed or reversed based on chart review and further discussion until consensus was reached.

### Patient characteristics

Patient demographic, clinical, and health utilization characteristics were obtained from administrative health insurance claims during the study period. Demographic and clinical characteristics included age, race and ethnicity (obtained during chart review), region, length of health plan eligibility in years, and comorbidity burden such as pediatric complex chronic condition (CCC) index [11], bone health issues (eg, fragility, osteoporosis, or vertebral fractures), impaired growth, puberty delay, and apnea (Additional file 1: Table S1). Healthcare utilization variables included inpatient hospitalizations; outpatient or emergency department (ED) visits; and PCP or specialist visits.

### Statistical analysis

Positive predictive value (PPV) and 95% confidence intervals (CI) were calculated using a 2 × 2 contingence table. The PPV for MD was calculated as the total number of confirmed cases in medical charts divided by the total number of patients with MD found in claims for which we obtained medical records. The percentage of specific MDs was calculated as total number of confirmed specific MD cases in medical charts divided by the total number of general MD cases in medical charts. Patient characteristics were summarized using basic descriptive statistics. All data were analyzed using SAS Enterprise Guide 7.1 (SAS, Cary, NC).

## Results

A total of 580 patients were identified from HIRD by the algorithm. Of those, 510 cases had either neurologist or PCP/pediatrician involved during MD care and 204 cases were current active fully insured members allowed for chart studies. Among the 204 cases for which medical charts were requested, charts for 109 patients were obtained and reviewed by the nurse abstractors (53.4%, Table 1).

**Table 1:** Patient Count

Criteria	n
Male patients with ≥2 office visits with diagnosis of hereditary progressive MD^a^ between 01/01/2013 and 12/31/2016 and < 18 years of age at time of first diagnosis	580
Involvement of either neurologist or PCP/pediatrician during MD care	510
Patients allowed for chart studies (e.g., current active fully insured members)	204
Charts obtained and abstracted	109

*MD* muscular dystrophy, *PCP* primary care physician

^a^ICD-9 359.1 or ICD-10 G71.0

Characteristics of the patients whose medical charts were obtained are summarized in Table 2. The average age was 12.6 years (SD 4.97). Overall, 74 (67.9%) patients had at least one pediatric complex chronic condition (other than neurologic and neuromuscular disease) as measured by modified CCC. 54 (49.5%) had cardiovascular conditions; 14 (12.8%) had respiratory conditions; 50 (45.9%) had bone-related issues; 11 (10.1%) had impaired growth; and 6 (5.5%) had puberty delay. The average length of health plan coverage was 2.8 years. Throughout their health coverage, 27 (24.8%) patients had more than one inpatient hospitalization and 38 (34.9%) had more than one ED visit. In general, the characteristics of patients whose medical charts were unobtainable did not differ from those whose medical charts were obtained (Additional file 2: Table S2).

**Table 2:** Characteristics of Patients Whose Medical Charts Were Obtained for Chart Validation

Patient Characteristics	Chart Obtained (*n* = 109)
Age, mean (SD)	12.6 (4.97)
Age category, n (%)	
< 4 (infants)	3 (2.8)
4–11 (children)	44 (40.4)
12–18 (adolescents)	47 (43.1)
≥ 19 (adults)	15 (13.8)
Race^a^, n (%)	
Caucasian/White	34 (31.2)
Black	0 (0.0)
Asian/Pacific Islander/native	3 (2.8)
Not documented	72 (66.1)
Ethnicity^a^, n (%)	
Hispanic	3 (2.8)
Non-Hispanic	15 (13.8)
Not documented	91 (83.5)
Census Region, n (%)	
Northeast	17 (15.6)
Midwest	30 (27.5)
South	32 (29.4)
West	30 (27.5)
Clinical history (claims)	
Length of medical enrollment in years, mean (SD)	2.8 (1.23)
Modified pediatric CCC^b^, mean (SD)	1.2 (1.25)
Modified pediatric CCC category, n (%)	
0	35 (32.1)
1 or 2	58 (53.2)
≥ 3	16 (14.7)
Any pediatric CCC^c^, n (%)	
Cardiovascular	54 (49.5)
Respiratory	14 (12.8)
Gastrointestinal	10 (9.2)
Metabolic	4 (3.7)
Hematologic or immunologic	3 (2.8)
Renal and urologic	3 (2.8)
Other congenital or genetic defect	43 (39.4)
Malignancy	4 (3.7)
Bone health issue, n (%)	50 (45.9)
Impaired growth, n (%)	11 (10.1)
Puberty delay, n (%)	6 (5.5)
Apnea, n (%)	4 (3.7)
Medical utilization history (claims), n (%)	
Any hospitalization	27 (24.8)
Any ED visit	38 (34.9)
Any outpatient visit	109 (100.0)
PCP visits	106 (97.2)
Specialist visits	109 (100.0)
Cardiologist	75 (68.8)
Pulmonologist	54 (49.5)

*CCC* complex chronic condition, *ED* emergency department, *PCP* primary care physician, *SD* standard deviation

^a^Obtained from medical chart

^b^Modified pediatric CCC counted the number of chronic conditions other than neurologic/neuromuscular diseases

^c^Neonatal as a pediatric CCC is not shown since no children were in that subcategory

The PPV of the case-identifying algorithm for MD was 95% (95% CI 88–98%, Table 3). Of the 103 confirmed MD cases, 87 patients (85%) had Duchenne or Becker MD (95% CI 76–91%); 76 patients (74%) had Duchenne MD (95% CI 64–82%), and 11 patients (11%) had Becker MD (95% CI 5–18%). Other less common types of MD included limb-girdle MD (4 patients), facioscapulohumeral MD (3 patients), Emery-Dreifuss MD (2 patients), congenital MD – unknown type (2 patients), progressive and/or hereditary MD – unknown type (2 patients), myotonic MD type 1 (1 patient), myotubular MD (1 patient), and Ulrich MD (1 patient).

**Table 3:** Summary of Validation Results – Positive Predictive Values

Diagnosis Confirmed in Medical Chart	n	Confirmed Cases	PPV % (95% CI)
Muscular Dystrophy	109	103	95 (88–98)
Duchenne or Becker MD	103	87	85 (76–91)
Duchenne	103	76	74 (64–82)
Becker	103	11	11 (5–18)

*CI* confidence interval, *MD* muscular dystrophy, *PPV* positive predictive value

### Discussion

The results of this study demonstrate that the case-finding algorithm can accurately identify patients with MD, primarily Duchenne MD, within a large administrative database. There was only one other study by Soslow et al. that established a claim-based algorithm to identify patients with Becker and Duchenne muscular dystropy. They reported PPV of 77%, but one limitation of the study was that they only looked at hospital encounter data, thus potentially missing a big proportion of patients with MD [12]. Our study was strengthened by the ability to capture events across physician office visits and hospital encounters. Our algorithm, which was constructed using a few items that are easily accessible from claims, achieved a PPV of 95% for MD and 85% for Becker and Duchenne MD. A deeper look into the types of MDs shows that majority of the cases were Duchenne MD (74%), followed by Becker (11%) and other MD types, consistent with known etiologies.

The algorithm achieved similar PPV for Becker and Duchenne MD when compared to the algorithm in Soslow et al.’s study. Yet, the two studies had employed different strategies to optimize the specificity of algorithms. In Soslow et al. study, patients with a change in primary diagnosis to a different 359.x code were excluded. An additional set of clinical characteristics was used to exclude MD other than Becker and Duchenne MD (e.g., patients with early mortality, in ventilatory support, or cardiovascular disease at a young age). Our algorithm required at least two office visits with MD diagnosis because it is widely known that using only one ICD diagnosis code to identify cases could introduce inaccuracy and false positives. Supporting evidence from previous studies has shown that use of two or more ICD diagnosis codes improves the PPV of case-finding algorithms [9, 10].

Using longitudinal claims data, the study also depicted the characteristics of patients with MD. Progressive muscular damage and degeneration in patients with MD manifests in muscular weakness, motor delays, respiratory impairment, and cardiomyopathy. As the disease progresses, the susceptibility to respiratory infection, respiratory compromise, and cardiomyopathy increase. These were confirmed in our study population: the prevalence of cardiovascular diseases increased from 0% among patients younger than 4 years to 67% among patients age 19 years and older. Additionally, the prevalence of respiratory disease increased from 0% among patients younger than 4 years to 27% among those age 19 years and older (data not shown). The overall rate of cardiovascular disease was much higher in our study (49.5% vs. 29.7% in Soslow et al. study), mostly likely due to their exclusion of patients with cardiovascular disease in the young age. While Soslow et al.’s study primarily focused on cardiovascular morbidity, our study also looked at the bone health and endocrine-related comorbidities, which are common in patients with MD and exacerbated by glucocorticoid treatment [5]. We observed that nearly half of our study population had bone-related conditions, and more than 10% had endocrine-related conditions. Using available claim information throughout their health plan coverage period, we were able to capture disease burden as well as healthcare utilization.

Anticipated emergence of genetic and molecular therapies, advances in rehabilitation therapies, and the invention of non-invasive prenatal testing for MDs spark many questions in the care of patients with MD, such as optimum timing for initiation of new therapies and optimal, personalized treatments [5, 6]. Given the small patient pool available, clinical trials will be challenged recruiting populations to investigate these questions. Observational studies, such as those using claims data, have a great amount of utility in advancing the knowledge base of MD care. Claims data reflect the comorbidities, treatment patterns, and the safety and effectiveness of therapies in typical patients seen in real-world settings, providing insight into the actual burden of Duchenne MD.

The study has a few limitations. Firstly, the ICD9/10 diagnosis code available at the time of this study was a single code for general MD and it did not differentiate subtypes. Since claims do not contain adequate clinical information, our case-finding algorithm was unable to distinguish between subtypes. Specific ICD-10 codes for Duchenne/Becker MD and facioscapilohumeral MD are planned to be introduced in October 2018 [13], which are expected to enhance future claims-based research by improving the homogeneity of study populations. Secondly, our study only sampled cases obtained from claims, and thus was unable to report negative predictive value and sensitivity. For rare conditions, the ideal chart review method is impractical given the large amount of medical charts to be reviewed. Alternatively, several sampling strategies to select code negative patients have been implemented by investigators and researchers, but they are prone to bias introduced by disproportional sampling of code positive and code negative patients [14]. Our algorithm picked up 48% of the patients with at least 1 MD diagnosis code. The sensitivity of the algorithm is unknown and needs to be evaluated with further research. Thirdly, the chart validation study sampled cases who were all likely had MD according to the algorithm. As the chart reviewers were not blinded to the study design, bias may have been introduced to move the PPV upward. Fourthly, our study characterized the co-morbidity burdens of patients with MD using claims data. Prevalence may vary by the type of diagnostic instruments used to define disease, however claims-based definition generally doesn’t include such information. Claim-based diagnosis may also subject to claim coding omissions or errors. Lastly, our data were drawn from commercially insured members who may not be representative of members under government or public insurance coverage.

## Conclusions

This study validated a case-finding algorithm that accurately identifies patients with MDs from a large administrative claims database. The algorithm can be used to facilitate epidemiological and health outcomes research.

## Additional files

Additional file 1: Code Lists Used to Define Baseline Comorbidity Burden; listing of ICD9/10 codes used to identify comorbidities at baseline. (DOCX 13 kb)

Additional file 2: Characteristics of Patients Whose Medical Charts Were Obtained Versus Those Whose Medical Charts Were Unobtainable; comparison of patient characteristics between patients whose charts were obtained vs patients whose charts were unable to be obtained. (DOCX 14 kb)

## Data Availability

The data supporting the findings of this study are available on request from the corresponding author. The data are not publicly available as they contain information that could compromise research participant privacy.

## Abbreviations

CCC: Complex chronic condition
CI: Confidence interval
CMS: Centers for Medicaid and Medicare Services
ED: Emergency department
HIRD: HealthCore Integrated Research Database
ICD 9/10: *International Classification of Diseases*, 9th and 10th Editions
IRB: Investigational Review Board
MD: Muscular dystrophy
PCP: Primary care provider
PPV: Positive predictive value
