# Supplementary material for: Chart validation of an algorithm for identifying hereditary progressive muscular dystrophy in healthcare claims
Source: BMC Med Res Methodol. 2019 Aug 9;19:174. doi: 10.1186/s12874-019-0816-7 (PMC6688201; doi:10.1186/s12874-019-0816-7)
Supplement: Supplementary file 1 — Code Lists Used to Define Baseline Comorbidity Burden; listing of ICD9/10 codes used to identify comorbidities at baseline. (DOCX 13 kb) [file 12874_2019_816_MOESM1_ESM.docx]

**Table S1. Code Lists Used to Define Baseline Comorbidity Burden**

| **Category** | **ICD-9 Diagnosis Codes (Start With)** | **ICD-10 Diagnosis Codes (Start With)** | **CPT Codes** | **HCPCS Codes** | **GPI Codes (Start With)** |
| --- | --- | --- | --- | --- | --- |
| Bone health issue^a^ | V5865, 7330, 805, 806, 8070, 8071, 8072, 8073, 808, 809, 810, 811, 812, 813, 814, 815, 816, 817, 818, 819, 820, 821, 822, 823, 824, 825, 826, 827, 828, 8290, 8291, V541, 77393, 77394, 77395, 77396, 77397, 2689, 2693, V5868 | Z7952, M80, M81, S22, S32, S72, S82, S42, S52, E559, E58, Z7983 |  |  |  |
| Impaired growth^b^ | 73391, 78340 | Z007, T38 |  | J2940, J2941, S9558 | 3010 |
| Puberty delay^c^ | 2572, 2590, 2571, 2572, V074 | E291, E300, E348, E349, Z79890 |  | J1071, S0189, J3121, J3145 | 23100030 |
| Apnea | 78603 | R0681 | 94774, 94775, 94776, 94777 | E0618, E0619 |  |
| ICD=International Classification of Diseases; CPT=Current Procedure Terminology; HCPCS=Healthcare Common Procedure Coding System; GPI=Generic Product Identifier  ^a^Included osteoporosis, vertebral compression fracture, leg bone fracture, arm bone fracture, chronic steroid use, vitamin D deficiency, calcium deficiency, and bisphosphonates  ^b^Included growth delay and human growth hormone supplementation  ^c^Included hypogonadism, delayed puberty, testosterone deficiency, and testosterone supplementation/replacement | | | | | |
