# Supplementary material for: Chart validation of an algorithm for identifying hereditary progressive muscular dystrophy in healthcare claims
Source: BMC Med Res Methodol. 2019 Aug 9;19:174. doi: 10.1186/s12874-019-0816-7 (PMC6688201; doi:10.1186/s12874-019-0816-7)
Supplement: Supplementary file 2 — Characteristics of Patients Whose Medical Charts Were Obtained Versus Those Whose Medical Charts Were Unobtainable; comparison of patient characteristics between patients whose charts were obtained vs patients whose charts were unable to be obtained. (DOCX 14 kb) [file 12874_2019_816_MOESM2_ESM.docx]

**Table S2. Characteristics of Patients Whose Medical Charts Were Obtained Versus Those Whose Medical Charts Were Unobtainable**

| **Patient Characteristics** | **Chart Obtained**  **(n=109)** | **Chart Not Obtained**  **(n=95)** | **P-value** |
| --- | --- | --- | --- |
| Age, mean (SD) | 12.6 (4.97) | 11.9 (4.48) | .224 |
| Age category, n (%) |  |  |  |
| < 4 years (infants) | 3 (2.8) | 0 (0.0) | .032 |
| 4 years to 11 years (children) | 44 (40.4) | 48 (50.5) |  |
| 12 years – 18 years (adolescents) | 47 (43.1) | 43 (45.3) |  |
| ≥19 years (adults) | 15 (13.8) | 4 (4.2) |  |
| Census region, n (%) |  |  |  |
| Northeast | 17 (15.6) | 8 (8.4) | .097 |
| Midwest | 30 (27.5) | 36 (37.9) |  |
| South | 32 (29.4) | 18 (18.9) |  |
| West | 30 (27.5) | 32 (33.7) |  |
| Unknown | 0 (0.0) | 1 (1.1) |  |
| Clinical history (claims) |  |  |  |
| Length of medical enrollment in years, mean (SD) | 2.8 (1.23) | 2.7 (1.30) | .602 |
| Modified pediatric CCC^a^, mean (SD) | 1.2 (1.25) | 1.2 (1.17) | .834 |
| Modified pediatric CCC category, n (%) |  |  |  |
| 0 | 35 (32.1) | 34 (35.8) | .510 |
| 1 or 2 | 58 (53.2) | 52 (54.7) |  |
| ≥3 | 16 (14.7) | 9 (9.5) |  |
| Any pediatric CCC^b^, n (%) |  |  |  |
| Cardiovascular | 54 (49.5) | 39 (41.1) | .225 |
| Other congenital or genetic defect | 43 (39.4) | 35 (36.8) | .702 |
| Respiratory | 14 (12.8) | 8 (8.4) | .310 |
| Gastrointestinal | 10 (9.2) | 6 (6.3) | .449 |
| Malignancy | 4 (3.7) | 2 (2.1) | .509 |
| Metabolic | 4 (3.7) | 16 (16.8) | .002 |
| Hematologic or immunologic | 3 (2.8) | 2 (2.1) | .766 |
| Renal and urologic | 3 (2.8) | 3 (3.2) | .864 |
| Bone health issue | 50 (45.9) | 45 (47.4) | .831 |
| Impaired growth | 11 (10.1) | 19 (20.0) | .046 |
| Puberty delay | 6 (5.5) | 9 (9.5) | .279 |
| Apnea | 4 (3.7) | 2 (2.1) | .509 |
| Medical utilization history (claims), n (%) |  |  |  |
| Any hospitalization | 27 (24.8) | 18 (18.9) | .317 |
| Any ED visit | 38 (34.9) | 24 (25.3) | .137 |
| Any outpatient visit | 109 (100.0) | 95 (100.0) | – |
| PCP visits | 106 (97.2) | 93 (97.9) | .766 |
| Specialist visits | 109 (100.0) | 92 (96.8) | .062 |
| Cardiologist | 75 (68.8) | 69 (72.6) | .550 |
| Pulmonologist | 54 (49.5) | 53 (55.8) | .373 |
| CCC=complex chronic condition; ED=emergency department; PCP=primary care physician; SD=standard deviation  ^a^Modified pediatric CCC counted the number of chronic conditions other than neurologic/neuromuscular diseases. ^b^Neonatal as a pediatric CCC is not shown since no children were in that subcategory | | | |
